# Large-Volume Lateral Lymph Node Metastasis Predicts Worse Prognosis in Papillary Thyroid Carcinoma Patients With N1b

**DOI:** 10.3389/fendo.2021.815207

**Published:** 2022-02-02

**Authors:** Luying Gao, Xiaoyi Li, Yu Xia, Ruifeng Liu, Chunhao Liu, Xinlong Shi, Yanjiao Wu, Liyuan Ma, Yuxin Jiang

**Affiliations:** ^1^ Department of Ultrasound, State Key Laboratory of Complex Severe and Rare Diseases, Peking Union Medical College Hospital, Chinese Academy of Medical Sciences and Peking Union Medical College, Beijing, China; ^2^ Department of General Surgery, State Key Laboratory of Complex Severe and Rare Diseases, Peking Union Medical College Hospital, Chinese Academy of Medical Sciences and Peking Union Medical College, Beijing, China

**Keywords:** papillary thyroid carcinoma, ultrasound, thyroid carcinoma, thyroid nodule, lymph node metastasis, lateral lymph node metastasis

## Abstract

We aimed to evaluate the relevance of large-volume lateral lymph node metastases (LLNMs) at risk of recurrence/persistence in papillary thyroid carcinoma (PTC) with LLNMs (N1b). This retrospective study included 448 PTC patients, who had positive LLNMs confirmed by histology and suspected of LLNMs by preoperative ultrasound. According to the number of pathological LLNMs, patients were divided into large-volume LLNM (number of LLNMs >5) and low-volume LLNM (number of LLNMs ≤5). Risk factors of recurrence/persistence in PTC patients with N1b were analyzed. Preoperative ultrasound features of PTC with large-volume LLNM were analyzed. For the patients with a mean follow-up of 44.0 months, the risk of recurrence/persistence was 25.1% in PTC patients with N1b. The recurrence/persistence rate was significantly higher in patients with large-volume LLNM than in patients with low-volume LLNM by multivariate analysis (37.3% vs. 17.1%; HR = 2.451, 95% CI 1.41–4.261, p = 0.001). The 3-year and 5-year recurrence/persistence-free survival for those with large-volume LLNM were 93.2% and 47.2%, respectively. Moreover, we found that multilevel suspected LLNMs and loss of fatty hilum were independent preoperative indicative factors of large-volume LLNM (OR = 6.239, 95% CI 3.547–10.977, p < 0.001; OR = 5.149, 95% CI 1.859–14.261, p = 0.002). In conclusion, multilevel suspected LLNM and loss of fatty hilum on ultrasound tended to be more common in patients with large-volume LLNM. PTC patients with large-volume LLNM are at a higher recurrent/persistent risk than those with low-volume LLNM. Large-volume LLNM may be used to stratify the risk of recurrence/persistence in PTC.

## Introduction

The incidence of papillary thyroid carcinoma (PTC) which accounts for 80%–85% of thyroid cancer in iodine sufficient areas has increased worldwide (1). The appropriate use of routine surgery can reduce disease recurrence and mortality, although they might lead to treatment-related complications (2). Therefore, it is crucial to balance the potential benefit versus possible adverse effects, to accurately choose the appropriate extent of surgery and other treatments. To investigate the risk stratification and help provide the appropriate treatment for a specific patient has become the cornerstone of individualized management of PTC by our and other teams’ results (1, 3, 4). Some PTCs are associated with poor clinical characteristics, such as significant cervical lymph node metastasis (LNM) or distant metastases (5, 6). Lateral neck metastasis is known as an independent risk factor of loco-regional recurrence, especially in patients older than 45 years (7–10). Moreover, large-volume lymph node metastasis (number of LNMs >5) has been shown to be a significant prognostic factor in the 2015 ATA guideline (1, 11–13). Central and lateral LNMs are considered to be two forms of local regional metastasis in PTC, since central lymph node metastasis (CLNM) has a lesser impact on survival (11). However, fewer studies have focused on large-volume lateral LNMs, and the utility of the number of lateral lymph node metastasis (LLNM) in the prognosis and nodal staging of PTC patients has not been comprehensively studied. Whether a significant number of LLNMs are more likely to remain or recurrence after surgery has not been confirmed.

Recent advances in ultrasound allow an accurate recognition of involved cervical compartments. In a recent meta-analysis, the sensitivity and specificity of ultrasound in detecting LLNM of PTC were 70% and 84%, respectively (14). Compartment-oriented lymph node (LN) dissection instead of the extensive radical LN dissection necessitates the close cooperation of a careful examination from radiologists. However, to our knowledge, no research had studied ultrasonographic features of cervical lymph nodes for large-volume LLNM. In this study, we assessed the prognosis and ultrasound features of cervical lymph nodes for large-volume LLNM in patients with PTC.

## Material and Methods

### Patients and Definitions

We retrospectively studied all 4,306 patients who underwent thyroidectomy at our hospital between December 2012 and April 2016. The following inclusion criteria were applied: (1) patients older than 18 years of age; (2) patients with PTC confirmed *via* surgical pathology; (3) patients who underwent thyroidectomy with central and lateral neck dissection; (4) patients with >5 lateral lymph nodes removed and lateral lymph node metastases confirmed *via* surgical pathology; and (5) patients with preoperative ultrasound suspected of having lateral lymph node metastasis. A total of 448 patients were included (**Supplementary Figure 1**). During follow-up, thyroglobulin (Tg), thyroid-stimulating hormone (TSH), and anti-Tg antibody levels were periodically measured in plasma, and ultrasound was periodically performed. If there was a suspicion of tumor recurrence/persistence, patients underwent whole-body 131-iodine scan, computed tomography (CT), magnetic resonance imaging (MRI), or 18-fluorodeoxyglucose positron emission tomography (PET). In the study, recurrence/persistence was defined as persistent or newly identified locoregional or distant metastases, which were reviewed by an experienced surgeon and an experienced radiologist in combination with Tg level (suppressed Tg >1 ng/ml or stimulated Tg >10 ng/ml) and Tg antibody status (rising Tg antibodies) (1, 15). Discrepancies were resolved through discussion.

### Data Collection

All ultrasound examinations were performed using a Philips iU 22 device (Philips Healthcare, Eindhoven, Netherlands), equipped with a 5–12-MHz linear-array transducer. The size (short axis and long axis), multifocality, shape, cystic appearance, fatty hilum, hyperechogenicity, calcifications, and vascularity of lymph nodes, were evaluated by ultrasound. The size, multifocality, and extrathyroidal extension of primary thyroid nodules were evaluated by ultrasound. Suspicious sonographic characteristics of cervical lymph nodes included loss of the fatty hilum, a rounded rather than oval shape, cystic change, hyperechogenicity, calcifications, and peripheral vascularity. In multifocal cases, the largest lymph node was analyzed. Lymph nodes were considered suspicious when one or more of the suspicious ultrasound findings were present (1). The ultrasound examinations were performed by radiologists, and the images of ultrasound were reviewed by an experienced radiologist who was blind to the patients’ clinical data.

The pathological data included tumor size, extrathyroidal extension, and the status of central and lateral LNs. In multifocal cases, the largest one was analyzed. Lateral cervical LNs included levels II, III, IV, and V lymph nodes. Central cervical lymph nodes included level VI and VII lymph nodes. The number of LNMs was calculated and analyzed with respect to the neck level. Large-volume LLNM referred to more than five metastatic lateral LNs. Low-volume LLNM referred to less than or equal to five metastatic lateral LNs. Large-volume CLNM referred to more than five metastatic central LNs.

### Statistical analysis

Categorical variables were presented as frequencies and analyzed using the chi-squared test. Quantitative data were presented as the mean ± standard deviations (SDs). For parametric data, an unpaired t-test was used to evaluate the differences between the two groups. The study analyzed the median and mean recurrence/persistence-free survival using the Kaplan–Meier method and log-rank testing. Univariate Cox regression was used to evaluate the association between each of the risk factors and the study’s recurrence/persistence. For multivariate analysis, the Cox proportional hazards model was used to assess the relationship between recurrence/persistence and variables. Based on the parameters from the statistically significant results of the χ2-tests, a multivariate logistic regression model was established to assess the correlations between sonographic features and large-volume LNM. All statistical analyses were performed with SPSS software version 19.0 (IBM, Armonk, NY, USA). Differences with p < 0.05 were considered statistically significant.

## Results

### Analysis of Risk Factors and Prognosis of PTC With Lateral Lymph Node Metastasis

**Figure 1:**
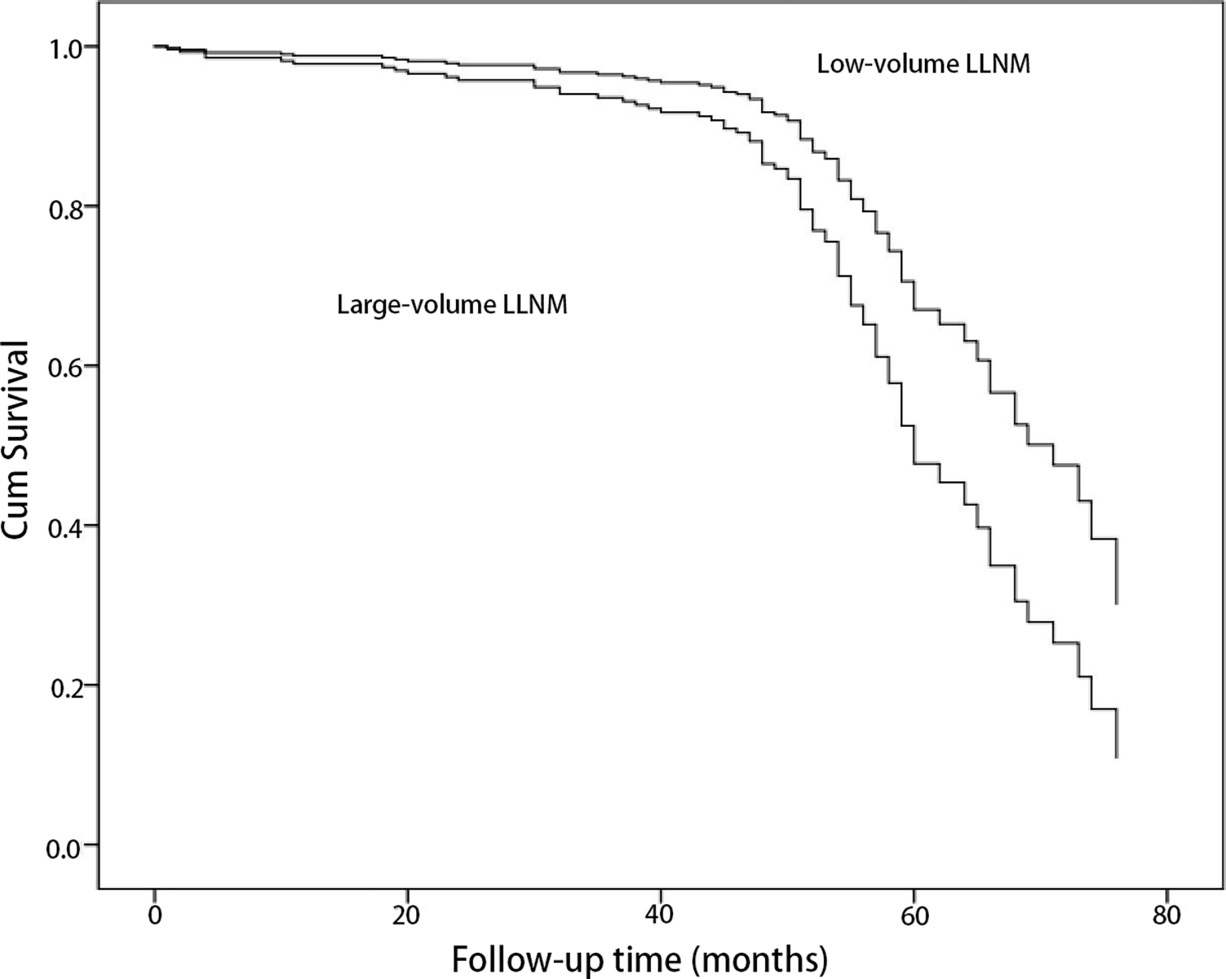
Comparison of cumulative survival of papillary thyroid cancer patients with metastasis to large-volume lateral lymph node and low-volume lateral lymph node by univariate Cox regression (p = 0.003).

**Figure 2:**
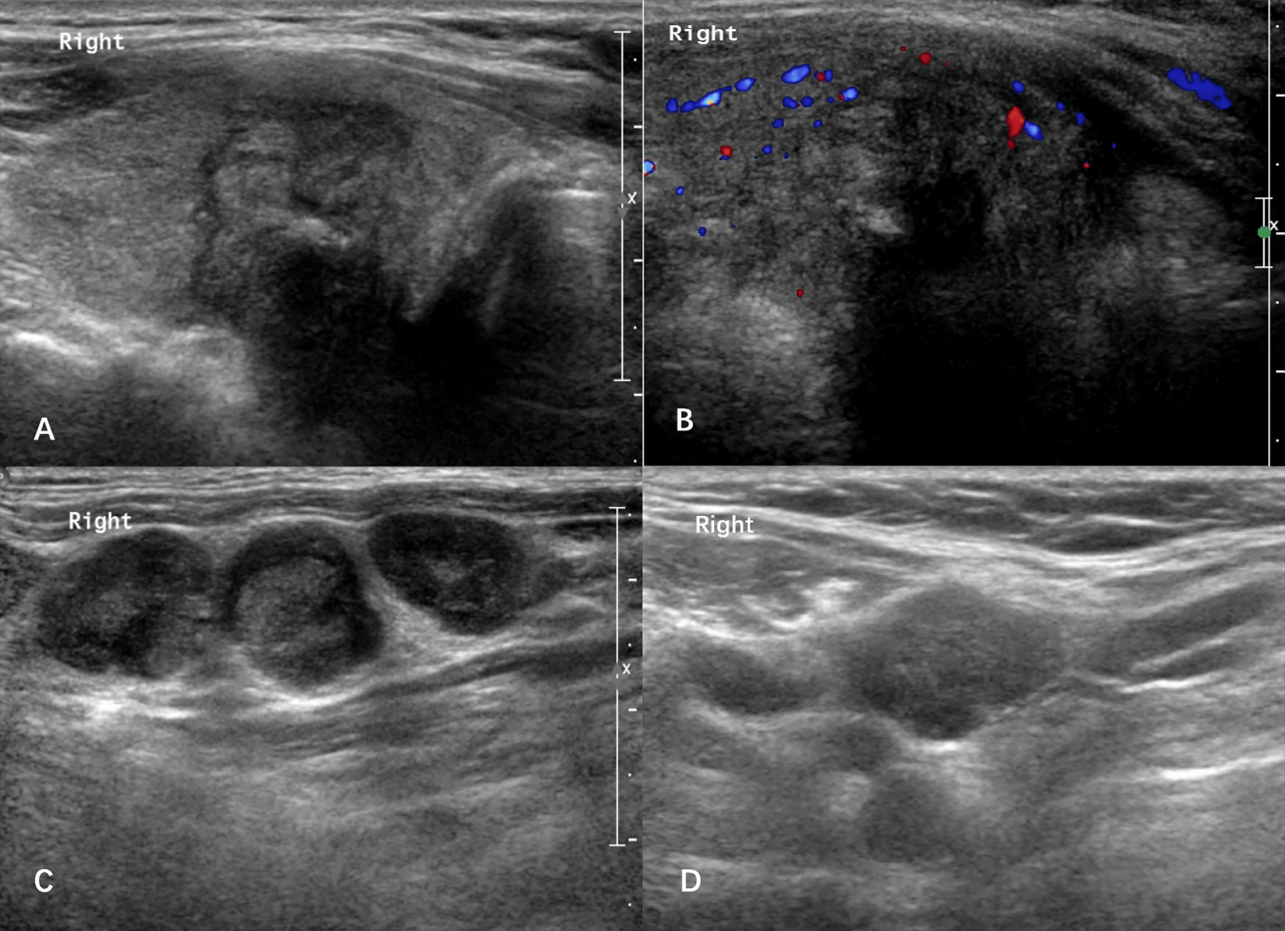
Case: a 61-year-old man was admitted due to hoarseness. Ultrasound showed there was a 2.6-cm solid thyroid nodule in the right lode **(A, B)**. The right-level III lateral lymph nodes showed rounded nodes with loss of the fatty hilum **(C)**. Histological pathology confirmed that the nodule was a papillary thyroid carcinoma with large-volume lateral lymph node metastasis in the right-level II/III/IV/V neck. After a follow-up period of 59 months, the patient developed the suspected cervical lymph node recurrence in the right neck with a thyroglobulin (Tg) level of 1.8 ng/ml **(D)**.

The correlation of risk factors with recurrence/persistence is shown in **Table 1**. The mean age of the patients at diagnosis with recurrence/persistence was 38.3 ± 12.1 years, and that of those without was 39.8 ± 11.2 years. Sex and age were not associated with recurrence/persistence (p = 0.057; p = 0.524). Large-volume CLNM correlated with recurrence/persistence (p = 0.01). 33.7% of patients with large-volume CLNM developed recurrence/persistence, compared with 18.8% of patients with low-volume CLNM. The recurrence/persistence rate was significantly higher in patients with large-volume LLNM than in patients with low-volume LLNM (37.3% vs. 17.1%, HR = 1.85, 95% CI 1.23–2.77, p = 0.003) (**Figures 1**, **2**). Other risk factors, such as multifocality, tumor size, extrathyroidal extension (ETE), and pathological CLNM, did not correlate with the prognosis of PTC.

**Table 1:** Risk factors of recurrence/persistence in PTC patients with lateral lymph node metastasis.

	Recurrence/persistence			
	Yes	No	HR (95% CI)	*p*-value
n (%)	97 (25.1)	290 (74.9)		
Male (%)	42 (30.2)	97 (69.8)	1.48 (0.99–2.22)	0.057
Age at diagnosis, mean ± SD, yr	38.8 ± 12.1	40.2 ± 11.5	0.99 (0.98–1.01)	0.524
<40 yr (%)	59 (32.2)	124 (67)	1.52 (1.00–2.31)	0.050
Multifocality (%)	77 (27.8)	200 (72.2)	1.34 (0.82–2.20)	0.240
Tumor size > 1 cm (%)	61 (26.4)	170 (73.6)	1.42 (0.94–2.15)	0.096
Pathological ETE (%)	60 (24.5)	185 (75.5)	1.05 (0.56–1.96)	0.880
Pathological CLNM (%)	94 (26.6)	259 (73.4)	3.15 (0.995–9.95)	0.051
Large-volume CLNM (%)	55 (33.7)	108 (66.3)	1.69 (1.13–2.54)	0.011
Large-volume LLNM (%)	57 (37.3)	96 (62.7)	1.85 (1.23–2.77)	0.003
Low-volume LLNM (%)	40 (17.1)	194 (82.9)		

LLNM, lateral lymph node metastasis; CLNM, central lymph node metastasis; ETE, extrathyroidal extension; yr, year; PTC, papillary thyroid cancer; CI, confidence interval; SD, standard deviation.

To identify independent factors associated with recurrence/persistence, variables were entered into multivariate Cox regression testing. Patients with large-volume LLNM were more likely to develop recurrence/persistence than those with low-volume LLNM (HR = 2.451, 95% CI 1.41–4.261, p = 0.001) (**Table 2**).

**Table 2:** Multivariate analysis for risk factors of recurrent/persistent status in PTC with lateral lymph node metastasis.

	β	SE	Wald	p	HR	95% CI
Male	0.31	0.291	1.133	0.287	1.364	0.77–2.414
Age < 40 yr	0.442	0.28	2.499	0.114	1.556	0.899–2.691
Multifocality	0.407	0.319	1.631	0.202	1.502	0.804–2.805
Tumor size > 1 cm	-0.045	0.288	0.024	0.877	0.956	0.544–1.682
Pathological ETE	0.387	0.278	1.932	0.164	1.472	0.853–2.539
Large-volume CLNM	0.5	0.284	3.097	0.078	1.648	0.945–2.876
Large-volume LLNM	0.897	0.282	10.101	0.001	2.451	1.41–4.261

LLNM, lateral lymph node metastasis; CLNM, central lymph node metastasis; CI, confidence interval; OR, odds ratio; PTC, papillary thyroid cancer; ETE, extrathyroidal extension; yr, year; SD, standard deviation.

The median and mean recurrence/persistence-free survival were 60.0 and 60.6 months for the PTC patients with large-volume LLNM, respectively. The median and mean recurrence/persistence-free survival were 69.0 and 65.5 months for the PTC patients with low-volume LLNM. The prognoses of PTC patients with large-volume LLNM were significantly worse than those with low-volume LLNM (p = 0.002) (**Table 3**).

**Table 3:** The median and mean recurrence/persistence -free survival time of the PTC patients with large-volume lateral lymph node metastasis and low-volume lateral lymph node metastasis.

	Median survival time, months (95% CI)	Mean survival time, months (95% CI)
Large-volume LLNM	60 (56.67–63.33)	60.59 (57.47–63.71)
Low-volume LLNM	69 (63.01–74.99)	65.48 (62.82–68.14)

PTC, papillary thyroid cancer; LLNM, lateral lymph node metastasis.

### Clinical Courses of PTC Patients With Lateral Lymph Node Metastasis

Of the 448 patients, 387 patients (86.4%) were followed up. The mean follow-up periods were 44.0 ± 19.1 months. The 3- and 5-year recurrence/persistence-free survival was 95.3% and 57.9%, respectively. Recurrence/persistence was recorded in 97 patients (25.1%), including 90 patients (23.3%) in the regional lymph node, five patients (1.3%) in the thyroid operative bed, and one patient (0.3%) in the cervical soft tissue, and one patient (0.3%) developed distant metastasis in the lung.

In our series, patients with recurrence/persistence had significantly higher abnormal Tg values (defined as suppressed Tg >1 ng/ml or stimulated Tg >10 ng/ml or rising Tg antibodies) than those without (31/95, 32.6% vs. 25/274, 9.1%, p < 0.001). The mean serum Tg level in the patients with recurrence/persistence was greater than those without (3.34 ± 12.05 vs. 0.97 ± 3.65 ng/ml, p = 0.01).

The mean serum Tg levels of patients with large-volume LLNM and with low-volume LLNM were 3.10 ± 11.62 and 0.86 ± 3.45 ng/ml, respectively. Patients with large-volume LLNM had a higher Tg level than those with low-volume LLNM (p = 0.008). The mean serum Tg antibody levels of patients with large-volume LLNM and with low-volume LLNM were 94.60 ± 424.76 and 33.35 ± 70.87 IU/ml, respectively (p = 0.11).

### Clinical Courses of PTC Patients With Large-Volume Lateral Lymph Node Metastasis

The prevalence of large-volume LLNM was 4.1% in PTC patients and 39.5% in PTC patients with lateral lymph node metastasis (LLNM). For the PTC patients with large-volume LLNM, recurrence/persistence was found in 57 cases (37.3%), including 53 patients (34.6%) in the cervical lymph node, 3 patients (2.0%) in the thyroid operative bed, and one patient (0.7%) in cervical soft tissue. The 3- and 5-year recurrence/persistence-free survivals were 93.2% and 47.2%, respectively.

Twenty patients (38.6%) with large-volume LLNM had recurrence/persistence lesions in the primary site of LLNM. 62.5% of patients with low-volume LLNM had recurrence/persistence lesions in the primary site of LLNM. More patients with low-volume LLNM had recurrence/persistence lesions in the primary site of LLNM than those with large-volume LLNM (62.5% vs. 38.6%, p = 0.003).

### Ultrasonographic Features for Large-Volume Lateral Lymph Node Metastasis

Patients with larger tumors (>2 cm) had a significantly higher large-volume LLNM rate than those with smaller tumors (55.7% vs. 36.5%, p = 0.003). The patients with large-volume LLNM tended to involve more levels of LLNM on the US at the lateral neck compared to those with low-volume LLNM (76.2% vs. 33.6%, p < 0.001). Patients with large-volume LLNM had larger LN on the US than those with low-volume LLNM (short axis of LN, 0.93 ± 0.55 vs. 0.65 ± 0.33 cm, p < 0.001; long axis of LN, 1.98 ± 0.96 vs. 1.46 ± 0.71 cm, p < 0.001). Compared to low-volume metastatic LNs, large-volume metastatic LNs were more likely to have the following ultrasound features: multifocality suspected LLNM (46.1% vs. 21.2%, p < 0.001), loss of fatty hilum (42.8% vs. 28.2%, p < 0.001), cystic change (53.8% vs. 37.6%, p = 0.03), and peripheral vascularity (46.1% vs. 29.6%, p < 0.001). There was no significant difference between large-volume and low-volume lateral LNs in terms of the ultrasound characteristics of hyperechogenicity, calcifications, and LN L/S ratio (p > 0.05). Moreover, suspected CLNM on ultrasound (US) was correlated with large-volume LLNM (46.4% vs. 34.3%, p = 0.011) (**Table 4**).

**Table 4:** Ultrasonographic features for large-volume lateral lymph node metastasis.

	Low-volume LLNM	Large-volume LLNM	p-value
n (%)	271 (60.5)	177 (39.5)	
Tumor size, mean ± SD, cm	1.28 ± 0.72	1.58 ± 0.91	<0.001
>2 cm (%)	31 (44.3)	39 (55.7)	0.003
ETE on US (%)	19 (51.4)	18 (48.6)	0.248
Multifocality suspected LLNMs on US (%)	178 (53.9)	152 (46.1)	<0.001
Multilevel suspected LLNMs	91 (40.3)	135 (59.7)	<0.001
Loss of the fatty hilum (%)	210 (57.2)	157 (42.8)	<0.001
Hyperechogenicity (%)	11 (42.3)	15 (57.7)	0.06
Cystic change (%)	24 (46.2)	28 (53.8)	0.03
Calcifications (%)	113 (55.9)	89 (44.1)	0.09
Peripheral vascularity (%)	145 (53.9)	124 (46.1)	<0.001
LLN short axis, mean ± SD, cm	0.65 ± 0.33	0.93 ± 0.55	<0.001
LLN long axis, mean ± SD, cm	1.46 ± 0.71	1.98 ± 0.96	<0.001
LLN L/S	2.46 ± 1.04	2.35 ± 0.93	0.26
<2 (%)	91 (59.9)	61 (40.1)	0.65
Suspected CLNM on US (%)	103 (53.6)	89 (46.4)	0.011

LLNM, lateral lymph node metastasis; CLNM, central lymph node metastasis; LLN, lateral lymph node; US, ultrasound; LLN, lymph node; L/S, long/short; ETE, extrathyroidal extension; SD: standard deviation.

The results of the multivariate logistic regression analysis of the suggestive factors are shown in **Table 5**. Multilevel suspected LLNMs and loss of fatty hilum were found to be independent factors indicative of large-volume LLNM (OR = 6.239, 95% CI 3.547–10.977, p <0.001; OR = 5.149, 95% CI 1.859–14.261, p = 0.002).

**Table 5:** Multivariate analysis of the sonographic features of large-volume lateral lymph node metastasis.

	β	SE	Wald	p	OR	95% CI
Tumor size > 2 cm	0.658	0.337	3.823	0.051	1.932	0.998–3.737
ETE	-0.092	0.741	0.015	0.901	0.912	0.213–3.901
Multifocality suspected LLNMs	0.009	0.374	0.001	0.982	1.009	0.485–2.1
Multilevel suspected LLNMs	1.831	0.288	40.352	<0.001	6.239	3.547–10.977
Loss of the fatty hilum of LN	1.639	0.52	9.943	0.002	5.149	1.859–14.261
Cystic change of LN	0.583	0.384	2.303	0.129	1.792	0.844–3.807
Calcifications of LN	0.401	0.263	2.33	0.127	1.493	0.892–2.499
Peripheral vascularity of LN	0.361	0.289	1.563	0.211	1.434	0.815–2.525
LLN L/S < 2	-0.311	0.265	1.378	0.24	0.733	0.436–1.232
Suspected CLNM	0.426	0.26	2.688	0.101	1.532	0.92–2.55
Constant	-3.628	0.606	35.847	0	0.027	

LLNM, lateral lymph node metastasis; CLNM, central lymph node metastasis; LLN, lateral lymph node; LN, lymph node; CI, confidence interval; OR, odds ratio; ETE, extrathyroidal extension; L/S, long/short.

## Discussion

The reported overall recurrence/persistence rate of PTC ranged from 1.4% to 29.0% (7). Patients with lateral LNM are prone to have a higher recurrence/persistence rate. A previous study showed that patients with N2 stage (LN > 3 cm) were 6.18 times more likely to show lymph node recurrence than were N-negative patients. In our study, the risk of recurrence/persistence was 25.1% in PTC patients with LLNM (pN1b). The rate was comparable to a subsequent series by Ito et al. (16), in which the risk of recurrence was 25% in clinical N1b patients with PTC > 1 cm. A study also showed that the risk of recurrence increased to 30% in the patients that had clinical N1b disease (17). Compared with CLNM (N1a), the American Joint Committee on Cancer (AJCC) system also increases the risk of LLNM (N1b) disease, which may be due to that N1b is an indicator of more aggressive PTCs.

Some previous studies have demonstrated that the number of lymph node metastasis influenced the prognosis of thyroid cancer (11, 18–20). In a recent study of the SEER database, the metastasis lymph node number was significantly associated with both cancer-specific survival and overall survival in patients with differentiated thyroid cancer (11). Although the AJCC system did not use the number of LNMs to increase the risk of recurrence, five pathologically involved LNs have been shown to be a significant prognostic factor in the ATA risk stratification of recurrence (1). Compared with LLNM, CLNM has a lesser impact on survival. However, the utility of the lateral lymph node metastasis in the sub-nodal staging of PTC patients and stratifying of the patients by survival has not been shown. According to our results, recurrence/persistence was found in 37.3% of patients with large-volume LLNM. The recurrence/persistence rate was significantly higher in patients with large-volume LLNM than in patients with low-volume LLNM by univariate and multivariate analyses. The 3- and 5-year recurrence/persistence-free survivals for those with large-volume LLNM were 93.2% and 47.2%, respectively. Similarly, a previous study of patients with clinical N1b disease also showed that having 5 or more clinically apparent metastases independently affected disease-free survival, which may have a limited sensitivity since more than half of pathologically confirmed LNMs can be missed by preoperative clinical evaluation (18). Our results presented additional information indicating that the presence of large-volume LNM in the lateral neck (N1b) is important data for postoperative recurrence risk stratification. The findings may be useful for thyroid cancer recurrence classification modifying.

Several studies defined the disease recurrence of thyroid cancer as evidence of disease following a remission period of 1 or 2 years (7). The concept of PTC persistence or recurrence after the operation is still difficult to distinguish because of its indolent nature (15). The persistent or recurrent lesions in the study refer to new lesions or residual lesions found during the follow-up after surgery. 38.6% of patients with large-volume LLNM had recurrence/persistence lesions in the primary location of LLNM, and most of the patients (84.2%) had recurrence/persistence lesions in the lateral neck. Moreover, we found that more patients with low-volume LLNM had recurrence/persistence lesions in the primary site of LLNM than those with large-volume LLNM (62.5% vs. 38.6%). We speculate that it may be due to the patients with large-volume LLNM being more aggressive and prone to metastasis, or patients with low-volume LLNM having persistent lymph node lesions after the initial surgery.

Due to the deep location of central compartment LNs, it is difficult to identify features of these LNs on ultrasound. Preoperative neck ultrasound has a low sensitivity (48.1%) for detecting CLN metastasis. Ultrasound is the preferred screening technique in the preoperative assessment of lateral neck lymph nodes with a sensitivity of 70%–80% (14, 21). Accurate and reliable preoperative ultrasound knowledge of these lateral metastatic lymph nodes will allow an individualized surgical approach (22). We explored the differences in preoperative ultrasound features for predicting large-volume LLNM. In our investigation, multilevel suspected LLNMs and loss of fatty hilum of LN were independently associated with large-volume LLNM. Although the loss of fatty hilum on ultrasound was associated with large-volume LLNM, the overall interobserver agreement of loss of fatty hilum was not satisfied (κ = 0.51) (23). The PTC patients with multilevel suspected LLNMs may be considered to perform more aggressive subsequent treatment for the lateral lymph node.

Our study has several limitations, including the retrospective study design and the mean follow-up time of 44 months, which was not a long period. Moreover, the size of the pathological lateral lymph nodes was not assessed by postoperative pathology. Thirdly, as ultrasound examination is real-time and operator-dependent, and the ultrasound characteristic evaluation in the study may be influenced by the operators. Finally, in the study recurrence/persistence was defined as persistent or newly identified locoregional or distant metastases in combination with the Tg level and Tg antibody status, which was not confirmed through cytology. Future studies are needed to confirm and extend our results.

## Conclusion

PTC patients with large-volume lateral lymph node metastasis were at a significantly higher recurrence/persistence risk than those with low-volume lateral lymph node metastasis (at least in the short-term follow-up). Large-volume lateral lymph node metastasis may be considered in the nodal staging for the prognosis risk stratification of PTC patients. Since the patients with large-volume LLNM tended to involve multilevel suspected LLNMs and LNs with loss of fatty hilum, these preoperative ultrasound findings may be useful for identifying patients at higher large-volume LLNM risk.

## Data Availability Statement

The raw data supporting the conclusions of this article will be made available by the authors, without undue reservation.

## Ethics Statement

The studies involving human participants were reviewed and approved by the ethics committee of Peking Union Medical College Hospital. Written informed consent for participation was not required for this study in accordance with the national legislation and the institutional requirements. Written informed consent was obtained from the individual(s) for the publication of any potentially identifiable images or data included in this article.

## Author Contributions

YX and XL conceived of and designed the study. LG was the major contributor in performing the analysis, writing the manuscript, and preparing the figures and tables. YJ, CL, and XL participated in the study design and edited the manuscript. RL, XS, LM, and YW participated in the verification of data, image quality verification, selection, and collection of samples. All authors contributed to the article and approved the submitted version.

## Funding

This study was funded by the Tibet Autonomous Region Science and Technology Project (XZ201901-GB-04), Tibet Autonomous Region Organization and Aiding Project (XZ2019ZR ZY05(Z)), and Peking Union Medical College Hospital (0104170).

## Conflict of Interest

The authors declare that the research was conducted in the absence of any commercial or financial relationships that could be construed as a potential conflict of interest.

## Publisher’s Note

All claims expressed in this article are solely those of the authors and do not necessarily represent those of their affiliated organizations, or those of the publisher, the editors and the reviewers. Any product that may be evaluated in this article, or claim that may be made by its manufacturer, is not guaranteed or endorsed by the publisher.

## Supplementary Material

Supplementary Figure 1 Flowchart of patient selection and inclusion in the study.
