# Supplementary figures and images for: Large-Volume Lateral Lymph Node Metastasis Predicts Worse Prognosis in Papillary Thyroid Carcinoma Patients With N1b
Source: Front Endocrinol (Lausanne). 2022 Feb 2;12:815207. doi: 10.3389/fendo.2021.815207 (PMC8847215; doi:10.3389/fendo.2021.815207)

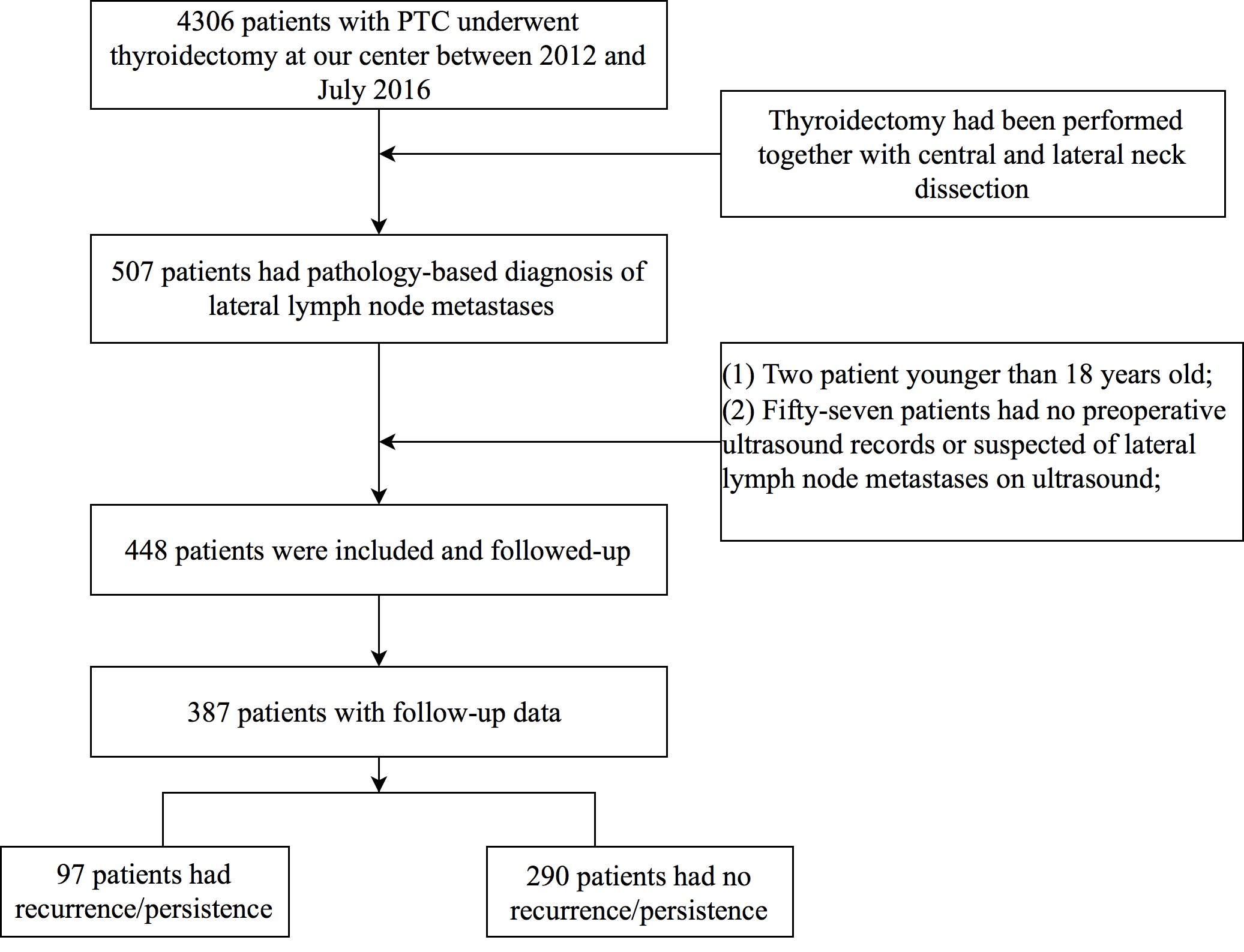

Supplement: Supplementary Figure 1 — Flowchart of patient selection and inclusion in the study. [file Image_1.tiff]
